# Magnetic Nanoparticles for Biomedical and Imaging Applications

**DOI:** 10.3390/ijms25115847

**Published:** 2024-05-28

**Authors:** Svetlana Konnova, Elvira Rozhina

**Affiliations:** Institute of Fundamental Medicine and Biology, Kazan Federal University, Kreml uramı, 18, 420008 Kazan, Republic of Tatarstan, Russia

Magnetic nanoparticles (MNPs) are a class of nanomaterials composed of metals such as cobalt, nickel, and iron with paramagnetic, ferromagnetic, or superparamagnetic properties [1]. This group includes materials such as transition metals (e.g., Fe, Co, Ni) and metal oxides (e.g., Fe_3_O_4_, g-Fe_2_O_3_) [2]. However, pure metal nanoparticles are chemically unstable in air and are easily oxidized; therefore, they are highly toxic [3]. In contrast, their oxides are less sensitive to oxidation and can produce a stable magnetic response [2].

The most widely studied nanoparticles with magnetic properties are iron oxide nanoparticles (SPIONs). Iron oxide nanoparticles exhibit unique properties such as superparamagnetism, high coercivity, a low Curie temperature, and high magnetic susceptibility [4]. Particularly important are particle properties that influence biodistribution within the body (e.g., size and surface hydrophobicity/hydrophilicity, and charge). Unlike bulk ferromagnetic materials, SPIO nanoparticles have no net magnetic moment before being placed in an external magnetic field [5].

Work on the development of new nanoparticles with magnetic properties continues. A search is being conducted all over the world to expand the areas of application of magnetic nanoparticles. The most fruitful application fields of nanomaterials are the use of magnetic nanoparticles in biomedical research and imaging. Therefore, the purpose of this Special Issue is to present new data on the use of magnetic nanoparticles in biomedical and imaging applications. One of the manuscripts submitted for this Special Issue aims to improve the properties of magnetite nanoparticles by incorporating zinc and/or cerium ions into its crystal lattice. Biological evaluation showed that such incorporation led to the emergence of antioxidant, anticancer, and antimicrobial properties in the nanostructures, with the effectiveness depending on the type and concentration of ions used for replacement [6].

Drug carriers are expected to have the following properties: biocompatibility, ease of surface modification, high encapsulation efficiency, long shelf life, and cost-effectiveness. Iron oxide nanoparticles have all these properties. Chan et al. developed a magnetic hyperthermia fluid based on FePt nanoparticles with cetyltrimethylammonium bromide (STAB)-modified kaolinite. These particles turned out to be promising for loading the cytostatic drug doxorubicin and can be used for the targeted delivery of drugs [7,8].

Magnetic nanoparticles are widely used in biomedical applications: magnetic hyperthermia [9], tissue repair, bacterial infection control, and use in dentistry, cardiology, neurology, and radiology. Other research articles provide insight into the use of magnetic nanoparticles in imaging applications.

The most important application of magnetic nanoparticles is in magnetic resonance imaging (MRI). MRI provides excellent in vivo soft tissue imaging capabilities with high spatial (<1 mm) and temporal resolution, and high contrast. However, the main limitation of MRI is its low sensitivity. To overcome this disadvantage, signal enhancement techniques such as the use of contrast agents are used to provide greater signal intensity, which generates higher contrast from the surrounding tissue. Thus, in MRI, SPIONs are used as a negative contrast agent to increase image contrast. Of particular interest are magnetic particles, namely, SPIO nanoparticles, consisting of maghemite (γ-Fe_2_O_3_), magnetite (Fe_3_O_4_), and other metallic ferrites [10]. SPIONs have been used orally to visualize the gastrointestinal tract [11], detect focal lesions in the liver and spleen [12,13], and to separate metastatic and benign lymph nodes [14].

We hope that this Special Issue provides some insight into the use of materials with magnetic properties in biomedical and imaging applications and presents useful new data in these areas.
